# Physical activity and risk of cholelithiasis: a narrative review

**DOI:** 10.3389/fmed.2024.1485097

**Published:** 2024-12-16

**Authors:** Zhen Ye, Jinkun Xie, Xiao Ni, Jiyong Yang, Jiong Li, Yujun Xuan, Honggang Gu

**Affiliations:** Department of General Surgery A, Longhua Hospital Shanghai University of Traditional Chinese Medicine, Shanghai, China

**Keywords:** physical activity, cholelithiasis, hazard ratio, risk, review

## Abstract

Cholelithiasis, commonly known as gallstone disease, poses a significant public health concern globally, with a myriad of risk factors contributing to its development. Among these, lifestyle factors, particularly physical activity, have garnered considerable attention for their potential role in modulating the risk of gallstone formation. This review aims to synthesize the current landscape of physical activity and the risk of developing cholelithiasis and identify knowledge gaps. To identify relevant articles, an independent author conducted a literature search using the PubMed database including keywords “cholelithiasis,” “gallstones,” and “physical activity” with no restriction on publication date. Cohort studies or Mendelian randomization analyses that reported physical activity and risk of gallstone disease were included in the current review. 15 articles were included in this review. The review highlights evidence suggesting a protective effect of regular physical activity against the development of gallstones. Results of Mendelian randomization analyses similarly demonstrated that physical activity remains independently causally associated with cholelithiasis. This review underscores the potential of physical activity as a modifiable risk factor for cholelithiasis, advocating for further research to establish definitive guidelines for prevention through lifestyle modification.

## Introduction

Cholelithiasis, commonly known as gallstone disease, is a prevalent condition characterized by the formation of solid particles, primarily composed of cholesterol and bilirubin, within the gallbladder or bile ducts (1). This condition affects a significant portion of the global population, with an estimated prevalence ranging from 10 to 15% in Western countries and up to 20% in certain regions of Asia and North America (2, 3). The presence of gallstones can lead to various complications, including acute cholecystitis, biliary colic, pancreatitis, and, in severe cases, life-threatening conditions such as gallstone ileus or cholangitis (4, 5).

While surgical intervention, particularly cholecystectomy (removal of the gallbladder), remains the primary treatment option for symptomatic cholelithiasis, there has been growing interest in exploring non-invasive approaches to manage and potentially prevent this condition (6). Physical activity has emerged as a promising intervention due to its potential benefits in modulating various risk factors associated with gallstone formation, such as obesity, dyslipidemia, and insulin resistance (7, 8).

The pathogenesis of cholelithiasis is multifactorial, involving a complex interplay of genetic, environmental, and lifestyle factors (9). Obesity, particularly abdominal obesity, has been consistently identified as a significant risk factor for gallstone formation (10, 11). Excess body weight and adiposity contribute to alterations in bile composition, promoting cholesterol supersaturation and the subsequent precipitation of cholesterol crystals, which can lead to gallstone formation (3). Additionally, insulin resistance and dyslipidemia, often associated with obesity, further exacerbate the risk of cholelithiasis (10, 12).

Physical activity has been extensively studied for its potential to mitigate these risk factors and promote overall metabolic health (13). Regular physical activity has been shown to improve insulin sensitivity, reduce abdominal obesity, and favorably modulate lipid profiles, thereby potentially reducing the risk of gallstone formation (14, 15). Furthermore, exercise has been associated with improved gallbladder motility, which may facilitate the clearance of bile and prevent the stagnation that contributes to gallstone formation (16). Notably, patients with chronic inflammatory bowel disease present an interesting case study in the relationship between physical activity and cholelithiasis. Recent evidence suggests that inflammatory bowel disease patients tend to have significantly lower physical activity levels compared to the general population (17). Simultaneously, these patients show a higher prevalence of cholelithiasis (18). This observation provides additional support for the potential role of physical activity in gallstone disease development, particularly in high-risk populations.

Despite the potential benefits of physical activity in the context of cholelithiasis, the existing literature remains fragmented, with varying study designs, assessment protocols, and outcome measures. The purpose of this review is to summarize the available evidence on physical activity for preventing cholelithiasis from the PubMed literature. By critically evaluating the available studies, this review seeks to provide insights into the potential role of such interventions in the management and prevention of gallstone disease, as well as to identify gaps and limitations in the existing research to guide future investigations.

## Method

To identify relevant articles, an independent author conducted a literature search using the PubMed database: ZY used the keywords “cholelithiasis,” “gallstones,” and “physical activity” with no restriction on publication date. The detailed search strategy is shown in Table 1. The inclusion criteria were published English-language articles in which physical activity exposure and incidence of gallstones were outcomes. We restricted study types to observational studies and Mendelian randomization analyses. Scoping reviews/systematic reviews were not included because many of the individual articles they included were already included in this review.

**Table 1:** Search strategy for PubMed database.

NO.	Search strategy
1	“Cholelithiasis”[Mesh]
2	“Gallstones”[Mesh]
3	“Cholecystolithiasis”[MeSH]
4	“Gallstone*”[Title/abstract] OR “Gall Stones”[Title/abstract] OR “Biliary Calculi”[Title/abstract] OR “Gall Stone”[Title/abstract] OR “Common Bile Duct Calculi”[Title/abstract]
5	#1 OR #2 OR #3 OR #4
6	“Sedentary Behavior”[Mesh]
7	“Sedentary Behavior”[Title/Abstract] OR “Sedentary Behaviors”[Title/Abstract] OR “Sedentary Lifestyle”[Title/Abstract] OR “Sedentary Lifestyles”[Title/Abstract] OR “Physical Inactivity”[Title/Abstract] OR “Lack of Physical Activity”[Title/Abstract] OR “Sedentary Time”[Title/Abstract] OR “Sedentary Times”[Title/Abstract]
8	“Physical activity”[Title/Abstract] OR “physical activities”[Title/Abstract] OR “physically active”[Title/Abstract]
9	“Step per day”[Title/Abstract] OR “steps per day”[Title/Abstract] OR “step count”[Title/Abstract] OR “step/day”[Title/Abstract] OR “steps/day”[Title/Abstract] OR “step/d”[Title/Abstract] OR “steps/d”[Title/Abstract] OR “daily step”[Title/Abstract] OR “daily steps”[Title/Abstract]
10	“Exercise”[Mesh] OR “exercise”[Title/Abstract] OR “resistance training”[Title/Abstract]
11	#6 OR #7 OR #8 OR #9 OR #10
12	#5 AND #11

JKX and XN reviewed article titles, abstracts, and full papers to identify eligible articles based on the inclusion criteria. Initially, 199 articles were identified. After screening the titles and abstracts, 21 full-text articles were assessed for eligibility. Following the full-text review, 15 studies (12 cohort studies and three Mendelian randomization analyses) met the inclusion criteria and were included in this review.

### Physical activity and risk of cholelithiasis: evidence from cohort studies

**Figure 1:**
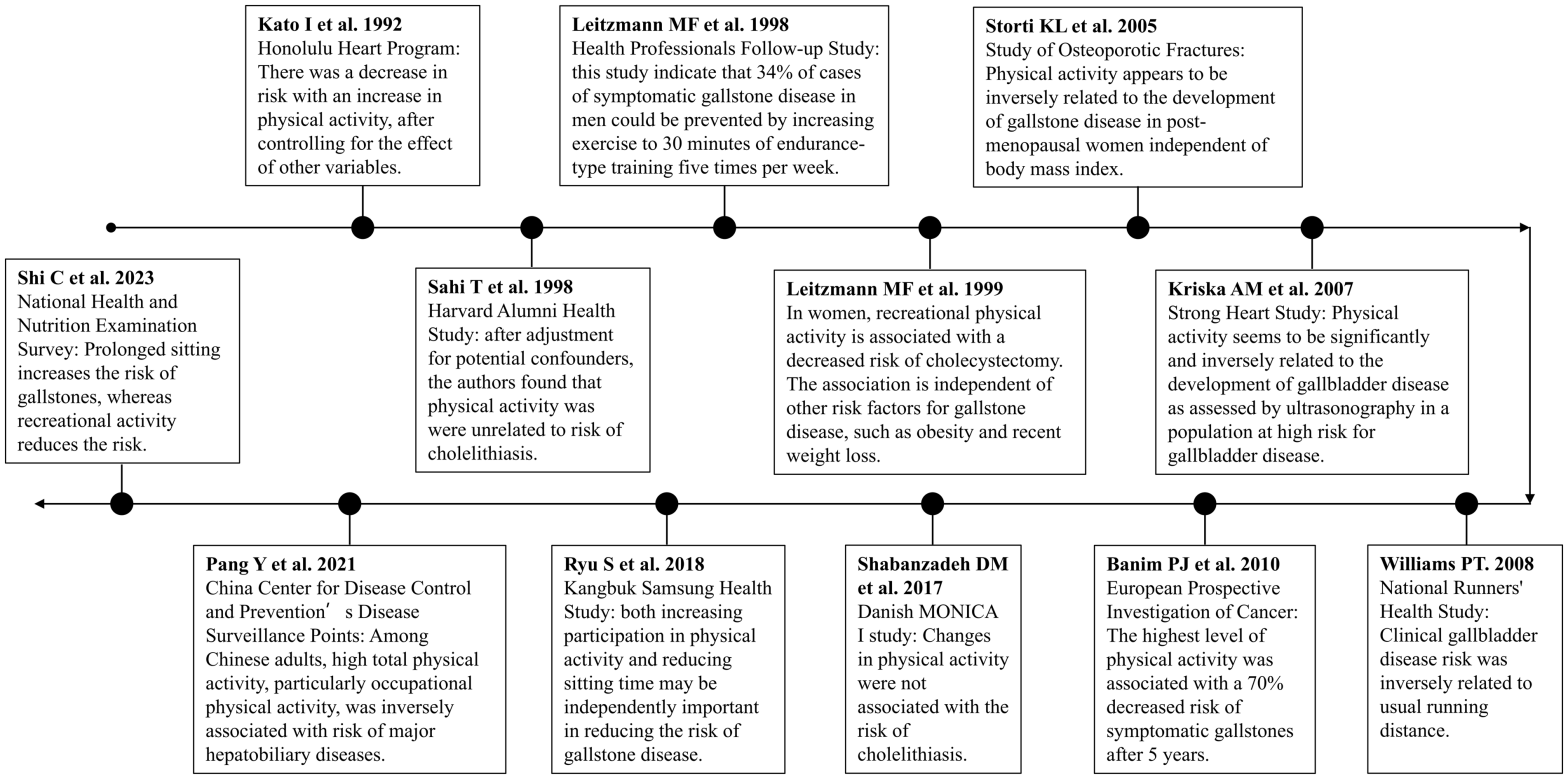
A timeline for physical activity and cholelithiasis.

Twelve cohort studies investigating the relationship between physical activity and risk of cholelithiasis were reviewed in this review. To better illustrate the development of research in this field, we created a timeline of physical activity and gallstone disease studies (Figure 1). This timeline demonstrates the evolution of research from early observational studies to more recent large-scale cohort studies and Mendelian randomization analyses. All included studies relied on self-reported physical activity to assess the exposure variable. The majority of these studies found an inverse association between physical activity and the risk of developing gallstones or symptomatic gallbladder disease.

Table 2 summarizes the key characteristics and findings of the 12 cohort studies included in this review. These studies were published between 1991 and 2023, with sample sizes ranging from 2,130 to 512,715 participants. Most studies were conducted in Western populations, including the United States (*n* = 8), Europe (*n* = 2), Korea (*n* = 1) and China (*n* = 1). Follow-up periods varied from 3.7 to 61 years.

**Table 2:** Characteristics of 12 included cohort studies.

Author (year) [Ref.]	Cohort name	Region	Cases/participants	Age range (median or mean)	% Male	Assessment of physical activity	Diagnosis of gallstone disease	Follow-up, years	Physical activity categories	Relative risk (95% CI)	Adjustments
Shi C et al. (2023) (30)	National Health and Nutrition Examination Survey	United State	1,214/11,970	47.93 ± 0.43	49.47	Simple question	Simple question	4	No	1.00	Age, sleep time, sitting time, NAFLD, trouble sleeping, ethnicity, marital, education, diabetes, walk or bicycle, work activity, smoke.
									Moderate	1.22 (0.91, 1.64)	
									Vigorous	0.50 (0.29, 0.87)	
Pang Y et al. (2021) (29)	China Kadoorie Biobank	China	11,170/512,715	52 ± 10.5	37.6	Physical activity questionnaire	ICD-10	10	<8.7 MET*h/day	1.00 (0.94, 1.06)	Age, education, household income, smoking, alcohol, self-rated health, diabetes, cardiovascular disease, respiratory disease, rheumatoid arthritis and sedentary leisure time
									8.8–14.2 MET*h/day	0.94 (0.89, 0.99)	
									14.3–21.8 MET*h/day	0.90 (0.86, 0.95)	
									21.9–33.1 MET*h/day	0.87 (0.83, 0.91)	
									> = 33.2 MET*h/day	0.86 (0.81, 0.90)	
									Per 4 units	0.98 (0.96, 0.99)	
Ryu S et al. (2018) (28)	Kangbuk Samsung Health Study	Korea	2,382/163,631	39.1 ± 7.5	53.9	International Physical Activity Questionnaire	Ultrasonography-diagnosed	3.7	Inactive	1.18 (1.05, 1.34)	Age, sex, center, year of screening examination, smoking status, alcohol intake, sleep duration, total calorie intake, BMI, history of cardiovascular diseases, history of diabetes and history of hypertension, homeostasis model assessment of insulin resistance, triglyceride and high-density lipoprotein-cholesterol.
									Minimally active	1.10 (0.98, 1.25)	
									health-enhancing physically active	1.00	
Banim PJ et al. (2010) (21)	European Prospective Investigation of Cancer (EPIC)-Norfolk	United Kingdom	290/25,639	59.0 ± 9.3	46.2	A self-administered questionnaire	ICD-10	14	Inactive	1	Sex, age, alcohol, and BMI
									Moderately inactive	1.18 (0.89, 1.58)	
									Moderately active	1.02 (0.73, 1.42)	
									Active	0.75 (0.50, 1.12)	
Sahi T et al. (1998) (23)	Harvard Alumni Health Study	United State	314/16,785	15–24	100	A self-administered questionnaire	Self-Reported, Physician-Diagnosed	61	<500 kcal/week	1	Age, BMI, BMI change between college and 1962/1966, calendar year, smoking
									500–999 kcal/week	1.21 (0.83, 1.76)	
									1,000–1999 kcal/week	1.01 (0.69, 1.47)	
									2000–3,499 kcal/week	1.33 (0.91, 1.95)	
									> = 3,500 kcal/week	0.72 (0.46, 1.13)	
Leitzmann MF et al. (1999) (24)	Nurses’ Health Study	United State	3257/60,290	40–65	0	A self-administered questionnaire	Self-Reported	10	0–1.6 MET*h/week	1	Age, parity, use of oral contraceptives, use of hormone-replacement therapy, history of diabetes mellitus, pack years of smoking, use of cholesterol-lowering drugs, use of thiazide diuretics, use of nonsteroidal anti-inflammatory drugs, intake of energy-adjusted dietary fiber, intake of energy-adjusted carbohydrates, intake of alcohol, intake of coffee, BMI, and weight change
									1.7–4.5 MET*h /week	0.95 (0.86, 1.06)	
									4.6–10.5 MET*h /week	0.92 (0.83, 1.03)	
									10.6–22.0 MET*h /week	0.83 (0.74, 0.93)	
									> = 22.1 MET*h /week	0.79 (0.71, 0.89)	
Storti KL et al. (2005) (20)	Study of Osteoporotic Fractures	United State	353/8,010	71.1 ± 4.9	0	A modified version of the Harvard Alumni Questionnaire	Self-Reported	7	<512 kcal/week	1.59 (1.11, 2.29)	Age, BMI, education, hormone use, diabetes prevalence, smoking status, alcohol use, parity
									512–1,189 kcal/week	1.57 (1.11, 2.23)	
									1,190–2,244 kcal/week	1.25 (0.87, 1.80)	
									>2,244 kcal/week	1	
Leitzmann MF et al. (1998) (19)	Health Professionals Follow-up Study	United State	828/45,813	40–75	100	A self-administered questionnaire	Self-Reported, Physician-Diagnosed	9	0–2.7 MET*h /week	1	Age; history of diabetes mellitus; smoking; and intake of cholesterol-lowering drugs, thiazide diuretics, nonsteroidal anti-inflammatory drugs, alcohol, energy-adjusted dietary fiber, energy-adjusted carbohydrates, and BMI
									2.8–7.7 MET*h /week	0.88 (0.72,1.08)	
									7.8–16.5 MET*h /week	0.83 (0.68,1.02)	
									16.6–32.5 MET*h /week	0.72 (0.58,0.89)	
									> = 32.6 MET hour/week	0.63 (0.51,0.79)	
Williams PT (2008) (26)	National Runners’ Health Study	United State	166/29,110	44.9 ± 10.4	100	Running distances daily	Self-Reported, Physician-Diagnosed	7.74	<2 km/day	1.00	Age, race, education, pack-years of cigarette use, weekly intakes of meat, fish, fruit, and alcohol, parity, and BMI
									2–4 km/day	0.78	
									4–6 km/day	0.85	
									6–8 km/day	0.49	
									> = 8 km/day	0.73	
Williams PT (2008) (26)	National Runners’ Health Study		112/11,953	38.9 ± 10.1	0	Running distances daily	Self-Reported, Physician-Diagnosed	7.42	<2 km/day	1.00	
									2–4 km/day	0.94	
									4–6 km/day	0.81	
									6–8 km/day	1.14	
									> = 8 km/day	0.59	
Kato I et al. (1992) (22)	Honolulu Heart Program	United State	471/7,931	45–65	100	Physical activity index	ICD-10	9	<29.5 MET*h/week	1	Age, alcohol intake, BMI, diastolic blood pressure, height, pack-years of cigarettes, serum random triglyceride, serum uric acid, serum glucose, total calorie intake
									29.5–31.8 MET*h/week	0.9 (0.7, 1.1)	
									31.9–35.4 MET*h/week	0.7 (0.6, 1.0)	
									> = 35.5 MET*h/week	0.6 (0.5, 0.8)	
Kriska et al. (2007) (25)^a^	Strong Heart Study	United State	650/2,130	45–74	37.8	A physical activity questionnaire	Ultrasonography-diagnosed	4	-	β: −0.023	Age, sex, and body mass index
Shabanzadeh DM et al. (2017) (27)	Danish MONICA I study	Denmark	226/2,366	30–60	51.9	Simple question	Ultrasonography-diagnosed	10	No change^b^	1	baseline age (years), body mass index (kg/m^2^) and alcohol consumption (units/week) (baseline and change), and baseline social group
									Decline	0.66 (0.35, 1.24)	
									Increase	1.08 (0.65, 1.79)	

NAFLD, non-alcohol fatty liver disease; BMI, body mass index; ICD-10, international classification of diseases; MET, metabolic equivalent.

a The authors included the model with a physical activity continuum variable.

b Changes with age unit.

The majority of studies consistently reported an inverse association between physical activity and gallstone risk. Most studies adjusted for key confounding factors including age, sex, body mass index, diet, and other lifestyle factors.

Physical activity was primarily assessed through self-reported questionnaires, with varying definitions and categories of activity levels. The ascertainment of gallstone cases was based on medical records/ self-report with medical confirmation in most studies.

Several studies, such as those by Leitzmann et al. (19) and Storti et al. (20), reported a dose–response relationship, with higher levels of physical activity conferring a greater protective effect against cholelithiasis. However, a few studies, like the one by Banim et al. (21), did not find a significant association between physical activity and cholelithiasis risk. This inconsistency might be attributed to differences in study populations, follow-up durations, and methods of assessing physical activity.

In a cohort study published in 1992 by Kato et al. (22), the association between lifestyle factors and the risk of cholelithiasis was investigated among 7,831 Japanese-American men. After 152,831 person-years of observation and adjusting for covariates, an increase in physical activity was associated with a decreased risk of developing cholelithiasis. The authors also acknowledged the limitations of the study, such as reliance on medical records for case diagnosis and self-reported physical activity, which may introduce bias. Further prospective studies are needed to validate these findings.

Two articles published in 1998 further explored the association between physical activity and the risk of developing cholelithiasis in different populations but reported inconsistent results. Sahi et al. (23) conducted a 61-year prospective study among 16,785 alumni of Harvard University aged 15–24. After adjusting for potential confounders, the authors found no association between physical activity and the risk of gallstones. Using participants with <500 kcal/week as the reference group, the rate ratios for 500–999 kcal/week, 1,000–1999 kcal/week, 2000–3,499 kcal/week, and > =3,500 kcal/week were 1.21 (95% CI: 0.83–1.76), 1.01 (95% CI: 0.69–1.47), 1.33 (95% CI: 0.91–1.95), and 0.72 (95% CI: 0.46–1.13), respectively. Considering that the study recruited highly educated young individuals who are more aware of healthy lifestyles, these findings are particularly intriguing. In another study, Leitzmann et al. (19) followed 45,813 men aged 40 to 75 from 1986 to 1994. After adjusting for various confounders, participants with 2.8–7.7 metabolic equivalent (MET)-h/week, 7.8–16.5 MET-h/week, 16.6–32.5 MET-h/week, and > =32.6 MET-h/week had relative risks (RR) of 0.90 (95% CI: 0.74–1.10), 0.89 (95% CI: 0.73–1.09), 0.78 (95% CI: 0.64–0.98), and 0.72 (95% CI: 0.58–0.90) compared to those with 0–2.7 MET-h/week of physical activity per week, with a trend test *p* = 0.003. When comparing the extreme quintiles, the inverse relationship with risk for men under 65 was stronger (multivariate RR = 0.58 [95% CI: 0.44–0.78]) than for men aged 65 or older (RR = 0.75 [95% CI: 0.52–1.09]). In contrast, prolonged sitting was positively associated with the risk of asymptomatic gallstones. Compared to men who watched TV for less than 6 h per week, men who watched TV for more than 40 h per week had a higher risk of developing asymptomatic gallstones (RR for older men = 3.32 [95% CI: 1.51–7.27]; RR for younger men = 1.58 [95% CI: 0.38–6.48]).

Subsequently, in 1999, Leitzmann et al. (24) reported another significant finding in the *New England Journal of Medicine* using data from the Nurses’ Health Study. Over a 10-year follow-up period (1986 to 1996), compared to women in the lowest quintile of physical activity, those in the highest quintile had a multivariable RR of 0.69 (95% CI: 0.61–0.78) for gallbladder removal surgery. In contrast, prolonged sitting was independently associated with an increased risk of gallbladder removal surgery. Compared to women who spent less than 6 h per week sitting at work or driving, those who spent 41 to 60 h had a multivariable RR of 1.42 (95% CI: 1.06–1.89), and those spending more than 60 h had a multivariable RR of 2.32 (95% CI: 1.26–4.26). These associations remained even after controlling for weight and weight change. However, the lack of data on surgery rates among this specific population of women somewhat weakens the association.

In 2005, Storti et al. (20) analyzed data from 8,010 postmenopausal women in the Study of Osteoporotic Fractures (SOF; 1986–1988) and found that, after adjusting for age, body mass index, education, hormone use, diabetes prevalence, smoking status, alcohol use, and parity, compared to participants in the fourth quartile (>2,244 kcal/week), those in the first (<512 kcal/week), second (512–1,189 kcal/week), and third (1190–2,244 kcal/week) quartiles had a 59% (hazard ratio [HR] = 1.59 [95% CI 1.11–2.29]), 57% (HR = 1.57 [95% CI: 1.11–2.23]), and 25% (HR = 1.25 [95% CI: 0.87–1.80]) increased risk of developing gallstones, respectively. Dose–response analysis showed that for every 500 kcal/week increase in total physical activity, the risk of gallstones could be reduced by 6% (HR = 0.94 [95% CI: 0.90–0.98]). However, the study was limited to community-dwelling Caucasian volunteers, greatly limiting its generalizability.

Considering that previous studies did not include patients with asymptomatic gallstones in their case groups, Kriska et al. (25) reported a study in 2007 involving 3,143 residents aged 45–74 from 13 American Indian communities in the United States. During the follow-up period (1993–1995), the gallbladder disease status of the entire cohort was assessed via ultrasound. After adjusting for potential confounders, including body mass index, an increase in physical activity was inversely associated with gallbladder disease status. These results remained consistent after stratifying the data by gender but were significant only among non-diabetics (with no significant impact on diabetics). Notably, this study lacked the collection of dietary information, limiting adjustments for dietary factors.

Following up in 2008, Williams (26) utilized the National Runners’ Health Study to analyze the association between self-reported gallbladder disease and physical activity (daily running distance) among 29,110 male and 11,953 female runners. Over the follow-up periods of 7.74 ± 1.84 years for men and 7.42 ± 2.10 years for women, 166 men (0.57%) and 112 women (0.94%) were diagnosed with gallbladder disease by physicians. Multivariate analysis indicated that the risk of clinical gallbladder disease was significantly associated with running distance (men: *p* = 0.01; women: *p* = 0.008), attributing this to the leanness of long-distance runners. Nonetheless, the generalizability of this cohort’s results is questionable as male and female runners who regularly participate in this activity might differ from the general population in terms of genetics, socioeconomic status, psychology, and other health behaviors.

Due to the lack of detailed physiological measurements of physical activity (including energy expenditure and cardiorespiratory function) in earlier published studies, Banim et al. (21) followed 25,639 volunteers aged 40–74 years recruited into the European Prospective Investigation of Cancer, Norfolk (EPIC-Norfolk) for 5 years, during which 135 cases of symptomatic gallstones were reported. After adjusting for covariates, the most active group had a significantly lower risk compared to the inactive group (HR = 0.27; 95% CI: 0.12–0.61). This result was similar in both men and women. Compared to the inactive group, the Moderately active and Moderately inactive groups were also associated with a lower risk of symptomatic gallstones, although their HRs were not statistically significant and the effect size was smaller compared to the most active group. Furthermore, the authors’ analysis based on the population-attributable risk suggested that increasing the physical activity level by one category across the entire population could prevent 17% of symptomatic gallstone diseases within 5 years. Interestingly, in a 14-year follow-up analysis, after multivariate analysis, an “active” level of physical activity was not significantly associated with a reduced risk of gallstones (HR = 0.75; 95% CI: 0.50–1.12). Other levels of physical activity had no impact.

Despite age and female gender being repeatedly considered as determinants for gallstones, their underlying mechanisms remain unclear. Thus, in 2017, Shabanzadeh and colleagues (27) conducted a cohort study on a random sample of the general population aged 30–60 years (*N* = 2,366) aimed at exploring whether physiological, lifestyle, or reproductive hormone changes with age are associated with the occurrence of gallstones. Contradictorily, in the multivariate-adjusted model, compared to participants with no change in physical activity, those with decreased physical activity had a 34% lower risk of developing gallstones (HR = 0.66 [95% CI: 0.35–1.24]), while those with increased physical activity had an 8% higher risk (HR = 1.08 [95% CI: 0.65–1.79]), though these results were not statistically significant. Given the self-reported assessment of lifestyle variables, this may lead to non-differential misclassification bias, explaining the non-significant estimates.

To further investigate the association between prolonged sitting time and the risk of gallstones diagnosed via ultrasound, Ryu et al. (28) published the results of the Kangbuk Samsung Health Study in 2018. Over 486,376 person-years of follow-up, a total of 2,382 cases of gallstones were identified. Prolonged sitting and lack of exercise were significantly independently associated with an increased risk of ultrasound-diagnosed gallstones. Compared to sitting less than 5 h, the HRs for gallstones after multivariate adjustment for sitting 5–9 h and ≥ 10 h were 1.08 (95% CI: 0.97–1.21) and 1.15 (95% CI: 1.02–1.29), respectively. Compared to the group with enhanced physical activity, the multivariate-adjusted HRs for gallstones in the inactive and minimally active groups were 1.22 (95% CI: 1.08–1.38) and 1.13 (95% CI: 0.99–1.28), respectively, with a trend test *p* < 0.001.

Due to the lack of association between specific domains of physical activity and gallstone disease, Pang et al. (29) analyzed 460,937 participants from the China Center for Disease Control and Prevention’s Disease Surveillance Points system and national health insurance system. Over a 10-year follow-up period, a total of 22,012 cases of hepato-biliary diseases were recorded. Compared to participants with total physical activity <8.7 MET-h/day, the risk of developing gallstones for those with physical activities of 8.8–14.2 MET-h/day, 14.3–21.8 MET-h/day, 21.9–33.1 MET-h/day, and > =33.2 MET-h/day was reduced by 6% (HR = 0.94 [95% CI: 0.89–0.99]), 10% (HR = 0.90 [95% CI: 0.86–0.95]), 13% (HR = 0.87 [95% CI: 0.83–0.91]), and 14% (HR = 0.86 [95% CI: 0.81–0.90]) respectively. Moreover, for every 4-unit increase in total physical activity, the risk of gallstone disease was reduced by 2% (HR: 0.98 [95% CI 0.96–0.99]). Compared to previous studies, this result reports a larger and more diverse population, utilizing data on both occupational and non-occupational physical activities, and extensively adjusting for risk factors of hepato-biliary diseases, making these findings more credible.

Recently, Shi et al. (30) analyzed data from 11,970 participants in the 2018–2020 National Health and Nutrition Examination Survey, which indicated that the risk of cholelithiasis increases with prolonged sitting time (odds ratio [OR] = 1.03; 95% CI: 1.00–1.05). Conversely, the risk of gallstones decreases with an increase in recreational activities (OR = 0.50; 95% CI: 0.29–0.87).

### Physical activity and risk of cholelithiasis: evidence from Mendelian randomization analysis

Observational data are limited by confounding and reverse causality, leading to limited power to identify causal associations. Mendelian randomization is a powerful epidemiological tool that uses genetic variants as instrumental variables to assess the causal relationship between an exposure and an outcome, minimizing the potential for confounding and reverse causation (31). Mendelian randomization studies use genetic information as instrumental variables to implement causal relationships, and it can be regarded as analogous to a randomized controlled study (32).

To date, we retrieved three Mendelian randomization studies on physical activity and risk of gallstone disease from the PubMed database.

Qian et al. (15) extracted pooled gallstone data from the UK Biobank (7,682 cases and 455,251 non-cases) and FinnGen consortium (23,089 cases and 231,644 non-cases). Based on a two-sample Mendelian randomization analysis, the authors found that the UK Biobank study demonstrated a negative causal relationship between accelerometer-predicted “mean acceleration” physical activity based on genes and gallstone risk (OR = 0.93; 95% CI: 0.87–0.99); there was a negative causal relationship between “overall activity” physical activity predicted from accelerometers and the risk of gallstone disease (OR = 0.38; 95% CI: 0.17–0.84). In the Finnish Genetic Alliance data, accelerometer-based “mean acceleration” physical activity was negatively associated with gallstone disease (OR = 0.94; 95% CI: 0.90–0.97). However, for self-reported moderate-intensity physical activity, no causal association was found in either data.

In the Mendelian randomization analysis of Shi et al. (30), the team extracted the effect estimates of selected instrumental variables from the “finn-b-K11_CHOLELITH” dataset. From the final results, both univariate and multivariate results, time spent watching TV (multivariate: OR = 1.646; 95% CI: 1.161–2.333) and physical activity (multivariate: OR = 0.953; 95% CI: 0.924–0.988) have independent causal relationships with gallstones.

Recently, Chen et al. (33) conducted an outcome-wide Mendelian randomization investigation to further reveal the intricate associations between sedentary behavior, physical activity, and various gastrointestinal diseases. Chen et al. and colleagues also obtained pooled data from the UK Biobank study, the FinnGen study, and large consortia, and their results suggest that genetically compensated longer leisure screen time is associated with an increased risk of gallstone disease (OR = 1.32; 95% CI: 1.23–1.41; *p* = 3.95 × 10^−14^). The association persisted after adjustment for genetic predisposition to moderate-to-vigorous intensity physical activity. Moderate-to-vigorous intensity physical activity genetics were associated with a reduced risk of gallstone disease (OR = 0.61; 95% CI: 0.51–0.73; *p* = 7.04 × 10^−8^). After adjusting for genetically predicted leisure screen time, the association weakened, but the directionality remained. Multivariate Mendelian randomization analyses identified body mass index and type 2 diabetes mellitus as mediators of the association of leisure screen time and moderate-to-vigorous intensity physical activity with gallstone disease.

### Gaps in the literature and future research directions

Despite the growing body of evidence linking physical activity to a reduced risk of cholelithiasis, there are still several gaps in the literature that warrant further investigation. To date, the greatest limitation in the evidence linking physical activity with a reduced risk of gallstone disease is that all of the studies rely on self-reported physical activity and sedentary behaviors, which may be subject to recall bias and misclassification. Although researchers can rank participants based on their reports, this is not suitable for determining the actual amounts of physical activity and sedentary time. Self-reported data may not accurately capture the intensity, duration, and frequency of physical activity, potentially leading to underestimation or overestimation of the true effects (34, 35). Future research should aim to use objective measures of physical activity, such as accelerometers or pedometers, to more accurately assess the relationship between physical activity and cholelithiasis risk (36).

Another gap in the literature is the lack of data on the optimal type, intensity, and duration of physical activity for reducing cholelithiasis risk. While some studies have suggested that moderate-intensity activities such as brisk walking may be sufficient (24), others have found that more vigorous activities may be necessary to achieve a protective effect (20). Future studies should aim to clarify the dose–response relationship between physical activity and cholelithiasis risk, as well as identify the specific types of activities that are most beneficial.

Additionally, there is a need for more research on the potential mechanisms underlying the association between physical activity and cholelithiasis. While some studies have suggested that physical activity may reduce cholelithiasis risk by improving insulin sensitivity and reducing inflammation (37), the exact pathways remain unclear. Future studies should aim to elucidate the biological mechanisms through which physical activity may protect against cholelithiasis, as this could help to inform the development of targeted interventions.

Finally, there is a need for more research on the potential moderating effects of factors such as age, sex, and body mass index on the relationship between physical activity and cholelithiasis risk. Some studies have suggested that the protective effect of physical activity may be stronger in certain subgroups, such as women or individuals with a higher body mass index (38), but more research is needed to confirm these findings and identify other potential moderators.

### Potential mechanisms

Several biological mechanisms may explain the protective effect of physical activity against cholelithiasis. Firstly, from the perspective of metabolic pathways: physical activity improves insulin sensitivity and reduces insulin resistance. Regular exercise helps maintain healthy cholesterol metabolism and reduces biliary cholesterol secretion. Activity-induced weight control helps prevent cholesterol supersaturation in bile (37, 38). Secondly, biliary kinetics: physical activity enhances gallbladder contractility and promotes more frequent gallbladder emptying. This improved bile acid metabolism and circulation helps prevent gallstone formation (4). Thirdly, in terms of inflammatory pathways: exercise reduces systemic inflammation markers and modulates pro-inflammatory cytokine production. Regular activity improves immune system function, which may help prevent gallstone development (3). Finally, hormonal effects are also potential mechanisms. Exercise influences hormones that regulate bile composition and modulates cholecystokinin secretion. Activity-related changes in sex hormone levels may affect gallstone formation (5).

### Strength and limitation

This article provides an overview of the research landscape regarding the association between physical activity and the risk of developing cholelithiasis. This review has several limitations. First, we only included articles found on PubMed, which means we may have missed eligible studies published in other databases. Second, the exposure in the included studies was based on self-reported physical activity, which to some extent reduces the reliability of the findings. Third, given the potential heterogeneity and data availability, we did not conduct further quantitative analysis.

## Conclusion

In conclusion, the comprehensive review of the PubMed literature underscores a significant association between physical activity and risk of cholelithiasis. The majority of the studies analyzed suggest that engaging in regular physical activity, particularly moderate to vigorous intensity exercises, can help prevent the formation of gallstones.

However, it is important to note that the relationship between physical activity and cholelithiasis is complex and may be influenced by factors such as age, gender, body mass index, and dietary habits. Some studies have reported inconsistent findings, highlighting the need for further research to establish the optimal type, intensity, and duration of physical activity required to minimize the risk of gallstone formation.

Despite these limitations, the evidence presented in this review supports the incorporation of regular physical activity as a preventive measure against cholelithiasis. Healthcare professionals should encourage their patients to adopt an active lifestyle, alongside other lifestyle modifications such as maintaining a healthy diet and managing body weight, to reduce the risk of developing gallstones. Future research should focus on conducting well-designed, large-scale prospective studies to further elucidate the relationship between physical activity and cholelithiasis, as well as to identify the most effective exercise interventions for gallstone prevention.

## Summary

Several studies analyzed suggest that regular physical activity, particularly moderate to vigorous intensity exercises, can help prevent gallstone formation. However, the relationship between physical activity and cholelithiasis is complex and may be influenced by factors such as age, gender, body mass index, and dietary habits. Despite some inconsistent findings, the evidence supports the incorporation of regular physical activity as a preventive measure against cholelithiasis. Healthcare professionals should encourage their patients to adopt an active lifestyle, alongside other lifestyle modifications, to reduce the risk of developing gallstones. Further research is needed to establish the optimal type, intensity, and duration of physical activity required for gallstone prevention.
